# 

**Published:** 1884-12

**Authors:** 


WHOLE NUMBER, CCLXXI.

DECEMBER, 1884.

NUMBER 5, VOLUME XXIV.

LIBRARY S.G.O. INDEXED

### THE BUFFALO Medical and Surgical Journal.

EDITORS:

THOS. LOTHROP, M. D., A. R. DAVIDSON, M. D.,

PUBLISHED BY THE MEDICAL JOURNAL ASSOCIATION.

No. 8 WEST CHIPPEWA ST.

Entered at the Post-Office at Buffalo, N. Y., as Second-class Mail Matter.

